# Measuring the prediction of observed actions using an occlusion paradigm: Comparing autistic and non‐autistic adults

**DOI:** 10.1002/aur.2716

**Published:** 2022-04-06

**Authors:** Emma Gowen, Ellen Poliakoff, Hayley Shepherd, Waltraud Stadler

**Affiliations:** ^1^ Division of Neuroscience and Experimental Psychology, School of Biology, Faculty of Biology, Medicine and Health Sciences The University of Manchester, Manchester Academic Health Science Centre Manchester UK; ^2^ Technical University of Munich Department of Sport and Health Sciences Munich Germany

**Keywords:** action perception, action prediction, autism, occlusion paradigm, simulation

## Abstract

Action prediction involves observing and predicting the actions of others and plays an important role in social cognition and interacting with others. It is thought to use simulation, whereby the observers use their own motor system to predict the observed actions. As individuals diagnosed with autism are characterized by difficulties understanding the actions of others and motor coordination issues, it is possible that action prediction ability is altered in this population. This study compared action prediction ability between 20 autistic and 22 non‐autistic adults using an occlusion paradigm. Participants watched different videos of a female actor carrying out everyday actions. During each video, the action was transiently occluded by a gray rectangle for 1000 ms. During occlusions, the video was allowed to continue as normal or was moved forward (i.e., appearing to continue too far ahead) or moved backwards (i.e., appearing to continue too far behind). Participants were asked to indicate after each occlusion whether the action continued with the correct timing or was too far ahead/behind. Autistic individuals were less accurate than non‐autistic individuals, particularly when the video was too far behind. A trend analysis suggested that autistic participants were more likely to judge too far behind occlusions as being in time. These preliminary results suggest that prediction ability may be altered in autistic adults, potentially due to slower simulation or a delayed onset of these processes.

**Lay Summary:**

When we observe other people performing everyday actions, we use their movements to help us understand and predict what they are doing. In this study, we found that autistic compared to non‐autistic adults were slightly less accurate at predicting other people's actions. These findings help to unpick the different ways that social understanding is affected in autism.

## INTRODUCTION

Observing other people's movement plays a key role in identifying, predicting and understanding the actions of others (Becchio et al., 2012). For example, when we observe a person performing an everyday task, such as hanging out laundry, we can infer from their actions whether they are relaxed or impatient and which pile of clothes they may be reaching for. For autistic individuals, who have difficulties with communication and social interaction (American Psychiatric Association, 2013), action perception can be challenging. The current work focusses on action prediction, which is a less researched aspect of action perception in autism.

Action prediction involves observing one or more actions and making a prediction about the outcome, intention or continuity of the movement.There are a number of different elements that facilitate action prediction such as the kinematics of the action (e.g., speed, direction), the objects that the person is interacting with and the environmental context (Amoruso et al., 2016; McDonough et al., 2019; Stapel et al., 2012; Wurm & Schubotz, 2012). People are remarkably good at predicting unfolding actions from just the kinematics of the movement (Ansuini et al., 2015; Becchio et al., 2012), such as predicting the direction of badminton strokes (Abernethy & Zawi, 2007), the size of an object to be grasped (Ansuini et al., 2016) and the intentions of a person (e.g., whether they are making a cooperative or competitive action; Cavallo et al., 2016; Manera et al., 2011; McEllin et al., 2018; Sartori et al., 2011). One mechanism thought to underlie action perception is the perception‐action system, consisting of a number of brain areas within the frontal and parietal cortices which show activation during observation of, and execution of, movement (Hardwick et al., 2018). It has been proposed that the perception‐action system facilitates action prediction by simulating the observed action, so that an internal forward model of the expected sensory outcomes is compared with the observed movement (Friston et al., 2011; Rizzolatti & Sinigaglia, 2010; Wolpert et al., 2003).

A number of previous studies comparing autistic and non‐autistic individuals have investigated a range of tasks tapping into different levels of action perception, such as biological motion detection (e.g., detecting human motion within noise), action discrimination (e.g., discriminating features such as direction or speed of movement) and action identification or inference (e.g., processing the motion for a secondary purpose such as inferring about others intentionality, emotional states or actions) (Federici et al., 2020). While findings are mixed, two recent meta‐analysis have identified overall poorer performance for autistic individuals (Federici et al., 2020; Todorova et al., 2019) but that group differences are more apparent for tasks that involve using the motion for a secondary purpose. Action prediction tasks may fall as a mixture of the latter two categories as participants are often required to use the motion for the secondary purpose of discriminating aspects such as the end action, goal or continuity of the movement (e.g., whether the action has continued at the expected pace or not). To date, there are only a few studies that have examined action prediction ability in autistic individuals.

Chambon et al. (2017) tested how autistic adults use prior expectations and visual information to predict whether an actor was transporting or rotating a cube during a non‐social (one actor) or social (two interacting actors) task. Expectation was manipulated by increasing the probability of occurrence of one of the actions for a particular block of trials and availability of visual information through reducing the video clip duration and hence the kinematic information. For both groups, prediction ability increased with the availability of visual information and for the more likely action. However, the non‐autistic group showed a higher number of responses for the more likely action in the social compared to non‐social task, whereas responses for the autistic group were similar for both tasks, suggesting that only the non‐autistic group relied more on prior information in a social context. Amoruso et al. (2019) employed a similar paradigm where autistic and non‐autistic children were asked to observe videos of children grasping objects to either interact with another child or not and predict the action (e.g., “to eat” or “to give”). Implicit contextual priors were induced by associating the different actions with contextual cues (e.g., a particular color of tablecloth was more likely to be associated with a particular action). Following familiarization, children performed the test phase where they were asked to predict the actions with much shorter videos so the amount of kinematic information was reduced. There were no differences in action prediction ability during familiarization when visual kinematic information was high. However, in the test phase non‐autistic children showed better performance for actions associated with higher probability contextual cues, whereas autistic children did not demonstrate differences between the probability conditions, indicating that they made less use of the contextual priors. It should be noted that the contextual priors used in this study are somewhat artificial and do not reflect information about a person's actions based on real‐world experience. Therefore, conclusions around how autistic children use learnt priors to understand actions is limited.

Eye tracking studies examining whether participants make anticipatory eye movements toward action goals have shown that autistic individuals make similar predictive eye movements (Falck‐Ytter, 2010; Marsh et al., 2015; Ward et al., 2021). However, prior knowledge about the frequency of an action appears to have less effect on anticipatory eye movements in autistic compared to non‐autistic children and adults, with the latter group only showing increased predictive eye movements to the goal with trial repetition (Schuwerk et al., 2016).

Overall these results suggest differences in the way that autistic individuals use prior knowledge such as social context and frequency to predict actions. However, as highlighted earlier, some of these studies use contextual cues that are unrepresentative of real‐world experience limiting conclusions about how autistic individuals use prior knowledge. In contrast, action kinematics are a relevant aspect of prior knowledge built up though observation of real‐world events. However, previous work has not directly examined the contribution of kinematics to action prediction in autistic individuals and required participants to make predictions based on relatively obvious and repetitive kinematic information (e.g., grasping a cup from the side or top), rather than making predictions about the ongoing movement (e.g., direction, speed) across different actions. There is evidence for altered processing of action kinematics in autistic individuals, suggesting that the contribution of kinematics to action prediction deserves investigation in this group. For example, autistic children and adults imitate the goal rather than the kinematics of actions (Gowen et al., 2020; Wild et al., 2012), are impaired at recognizing vitality form (how an action is performed such as rude or gentle; Di Cesare et al., 2017; Rochat et al., 2013) and are less able to discriminate the goal of an action from grasp kinematics (Boria et al., 2009; Turi et al., 2017) compared to non‐autistic groups. Autistic individuals also appear less able to plan and execute movements during a cooperative task when the required action relies on using kinematic information to predict a person's action (Fulceri et al., 2018). Further research is warranted to more directly assess autistic individuals' ability to use kinematics to predict actions, particularly considering the important role kinematics play in reacting to and understanding others actions (Ansuini et al., 2015; Becchio et al., 2012).

One frequently used action prediction paradigm that relies on observation of kinematics is the occlusion paradigm (Graf et al., 2007; Springer et al., 2013). This is based on the naturally occurring situation where a moving person is temporally occluded from view (e.g., passing behind a tree), yet viewers are usually quite good at predicting when the person should come back into view by extrapolating the trajectory of the person into the future. In a typical occlusion paradigm, participants observe an action which at various points is occluded for a period of time by a black screen. During the occlusion period, the action may be allowed to continue at the normal pace or moved ahead or behind and once the action reappears the participant must indicate whether the action is “in time” or too far ahead/too far behind. During the occlusion period, participants must recreate an accurate internal representation of the action, likely using action simulation or visual extrapolation (Stadler, Springer, et al., 2012) mechanisms. It is thought that action prediction during occlusion paradigms relies on motor simulation processes based on evidence that action prediction ability is linked with motor experience (Stapel et al., 2016) and negatively impacted by disruption to motor cortical areas (de Wit & Buxbaum, 2017; Stadler, Ott, et al., 2012).

The main aim of the current study was to examine action prediction ability in autistic compared to non‐autistic adults, using the occlusion paradigm that requires participants to use kinematics to predict the progress of a person's movement when performing naturalistic actions. If action prediction based on kinematics is altered in autistic individuals, we would expect lower prediction accuracy compared to the non‐autistic individuals. Furthermore, if simulation processes are slower or faster, this might result in lower accuracy for too far behind or too far ahead continuations respectively. Such prediction timing biases were characterized by examining the % of in time responses for each continuation. As an example, if participants had slower simulation processes, one might expect a higher percentage of in time responses for the too far behind continuation due to greater confusion between in time continuations and those that are too far behind. As prediction during this task is thought to use action simulation processes involving cortical motor areas, we also measured motor coordination and motor imagery ability to perform secondary, exploratory analyses. Motor coordination issues are common in autistic people (Gowen & Hamilton, 2013) and we hypothesized that poorer motor coordination may be correlated with less accurate prediction. As motor imagery is thought to involve imagining (simulating) oneself performing an action (Kilteni et al., 2018; Ridderinkhof & Brass, 2015; although see O'Shea & Moran, 2017 for a critique) we hypothesized that prediction and motor imagery ability may also be correlated.

## MATERIALS AND METHODS

Stimuli and occlusion duration were based on previous published work (Stadler et al., 2011).

### Participants

Twenty autistic (12 Female) and 22 non‐autistic (11 female) participants (Table 1) matched on age, sex, handedness and full scale IQ were recruited through the lab database, Autism@Manchester mailing list, local support groups and volunteer advertisements. As no previous data were available for autistic participants this sample number was based on previous work examining action prediction using occlusion methodology in non‐autistic individuals that have used similar sample sizes (Diersch et al., 2012; Stadler, Ott, et al., 2012). A power analysis indicated a total sample of 40 participants was required to detect a medium‐large group effect size (Cohens *f* = 0.36; *p* < 0.05; Power = 0.8, 2 groups and five measurements), while a total sample of 32 would detect a small to medium interaction (Cohens *f* = 0.2; *p* < 0.05; Power = 0.8, 2 groups and five measurements; G‐Power 3). Effect sizes in previous biological motion tasks in autism appear to depend on the complexity of the task with small‐medium effects for simpler detection or recognition tasks and large effect sizes (*d* = 0.73–1.77) for tasks that require using the motion for a secondary purpose such as inferring emotions, intentions or actions (Federici et al., 2020). While the current occlusion paradigm does not require inferring emotions or intentions it does require using kinematic information for the secondary purpose of determining whether the action has continued at the expected speed, so one might expect larger effect sizes. For the correlations, a total sample size of 19 would detect a large effect size (Cohens *r* = 0.5; *p* < 0.05; Power = 0.8, one tailed).

**TABLE 1:** Participant demographics. Statistical comparisons between the two groups on age, full scale IQ (FSIQ), performance IQ (PIQ) and verbal IQ (VIQ) are shown in the bottom row

Autistic group	Age (years)	Sex	Hand	FSIQ	PIQ	VIQ	ADOS	Non‐autistic group	Age	Sex	Hand	FSIQ	PIQ	VIQ
1	28.0	F	R	109	102	114	10	1	30.6	M	R	134	125	136
2	35.3	F	L	128	125	125	13	2	30.3	M	R	82	74	93
3	35.0	F	R	128	120	130	7	3	19.4	F	R	118	118	114
4	28.6	M	L	144	132	145	7	4	34.2	M	R	133	132	126
5	29.1	F	R	111	135	98	6	5	34.4	M	R	123	108	132
6	32.0	F	R	122	135	106	7	6	42.2	M	R	139	134	134
7	38.4	M	R	108	116	99	8	7	25.0	M	R	137	129	135
8	24	M	R	111	121	99	7	8	35.9	F	R	128	128	122
9	29.3	F	R	121	108	130	10	9	20.1	F	R	125	119	123
10	30.0	F	R	116	110	117		10	22.6	F	R	113	111	111
11	18.7	M	R	102	103	100	12	11	30.4	M	R	116	107	120
12	34.0	F	R	131	127	128	18	12	22	F	R	126	118	128
13	24.4	F	R	110	117	103	12	13	24	F	R	89	83	99
14	42.3	M	L	125	129	114	8	14	23	F	R	132	118	137
15	41.9	F	R	119	126	109	8	15	37.3	M	R	95	91	99
16	30.0	F	R	132	118	138	13	16	26	F	R	102	97	105
17	37.2	F	R	103	111	94	13	17	36	M	R	78	75	86
18	40	M	L	133	133	129	10	18	20.6	M	R	120	109	127
19	36	M	R	94	93	96	11	19	27.6	F	R	107	103	110
20	27	M	R	108	104	110	14	20	20.6	F	L	118	117	115
								21	18.9	F	L	109	100	116
								22	42.7	M	R	102	106	97
Mean ± *SD*	32.1 ± 6.3			118 ± 12.7	118 ± 12.2	114 ± 15.3	28.4 ± 7.5		28.4 ± 7.5			114 ± 17.6	109 ± 17.2	116 ± 15.1
Group tests	*t* = −1.73, *p* = 0.09			*t* = −0.61, *p* = 0.54	*t* = −1.95, *p* = 0.06	*t* = 0.51, *p* = 0.61								

Abbreviation: ADOS, autism diagnostic observation schedule.

Autistic participants had been given a diagnosis of Autism Spectrum Disorder by outside clinical assessment which was confirmed using module 4 of the Autism Diagnostic Observation Schedule by a qualified researcher (Lord et al., 2000). Age, Full Scale and Performance IQ, measured using Wechsler Abbreviated Scale of Intelligence did not differ significantly between the two groups (Table 1). In keeping with reports of a high rate of co‐occurring conditions in autism, three autistic participants reported having anxiety, one reported Semantic Pragmatic Disorder, one a diagnosis of Attention Deficit Hyperactivity disorder, three a diagnosis of Developmental Coordination Disorder, one Post Traumatic Stress Disorder and Tourettes and one Obsessive Compulsive Disorder. All had normal or corrected to normal vision of 6/6, using reduced Snellens at 33 cm. Participants gave written informed consent and the study was approved by The University of Manchester Research Ethics Committee.

### Apparatus and stimuli

**FIGURE 1:**
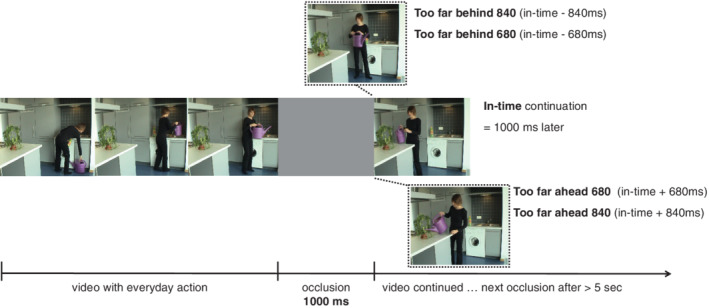
Trial layout. Each video was played for ~1 min and was transiently occluded by a gray rectangle for 1000 ms. Following the occlusion the video continued either in time, too far ahead by 680 or 840 ms or too far behind by 680 or 840 ms. Participants were required to indicate whether the action continued either in time, too far ahead or too far behind using key presses

Participants were seated at a wooden table in a lab cubicle facing a display screen (1920 × 1080 px, active viewing area—52.8 × 26.9 cm), which was positioned ~82.1 cm (±4.4 cm) from the participant's eyes. An EyeLink 1000 Plus eye‐tracker (SR research) collected eye movement data, using the desktop mounted, remote mode setting (head free‐to‐move) with a sampling frequency of 500 Hz and typical accuracy of 0.25–0.50°. Stimuli consisted of eight different videos of a female actor carrying out everyday actions: watering a plant, preparing a fried egg, filling a dishwasher, preparing coffee, changing the light bulb in a table lamp, putting‐up a poster, setting the table and folding laundry (Figure 1; example video can be found at Gowen et al., 2022). Each video lasted ~1 min and during this time the action was transiently occluded 2–6 times by a gray rectangle for 1000 ms at critical time points before a subgoal of the action was completed. The interval between each occlusion was at least 5 s. After an occlusion, the action sequence continued immediately.

During occlusions, the video was allowed to continue as normal or was moved forward or back in time. Five different continuation conditions were created (Figure 1). The video could be moved forward by 840 or 680 ms meaning that the action appeared to have proceeded too far in the future (too far ahead). The video could be moved backwards by 840 ms or 680 ms, meaning that the action appeared to have not proceeded enough (too far behind). Alternatively, the video was not manipulated and continued as normal (in time). These continuations were chosen following piloting to ensure the task would not be too difficult or easy for the participants, resulting in floor or ceiling effects (Diersch et al. (2012). The experiment was programmed in Presentation software (Neurobehavioral Systems).

### Procedure

Occlusion paradigm: This was composed of five parts, where parts 1–4 were training phases and part 5 was the experiment. In part 1, participants watched each video clip without disruption and completed a familiarity questionnaire, recording how familiar they were with performing each of the actions and associated sub‐actions on a scale of 1–10. In part 2, they watched a different video clip, but this time with occlusions that were preceded by a screen indicating the continuation type (too far behind, correct timing or too far ahead). In part 3, participants were trained on the task in a practice run consisting of 13 occlusions. Actions were occluded just as in the main experiment and participants were asked to indicate whether the action continued:

1. In time: video continues to play after the occlusion as if the video continued to play at normal speed behind the occlusion
2. Too far behind: video continues to play after the occlusion as if the video was paused during the occlusion and is too far behind
3. Too far ahead: video continues to play after the occlusion as if the video was sped up during the occlusion and is too far ahead

Participants made their choice by pressing keyboard buttons (1 = too far behind; 2 = in time;

3 = too far ahead) using three fingers of their dominant hand as quickly and accurately as possible. They received feedback after each occlusion consisting of smileys for correct/incorrect responses and a circle with missing face when response was missing). In part 4, participants trained using the “real experiment” situation which was identical to part 3 but without feedback. Parts, 2, 3 and 4 showed a video of the female actor hanging out clothes that was not used in the main experiment.

For the experiment phase (part 5), the eight videos were repeated in two blocks consisting of 36 occlusions each and separated by a break. There were a total of 72 occlusions (24 coherent and 12 for each of the four continuation conditions). The order of the videos was pseudo‐randomized but the occlusions were always presented at the same position within each particular video. Eye movements were tracked during the experiment phase.

Control task: At the end of the experiment, participants performed a control task to confirm their ability to use the keyboard and follow task instructions. They watched 32 short video clips involving sections of the videos shown in the main experiment. At a random onset after video‐start, 25 frames (corresponding to 1 s) were cut out of 24 of the videos, creating the effect of a visibly jumpy or jerky movement. Participants were instructed to detect when a frame was cut‐out of a video producing a noticeable jump and respond as soon as possible by pressing button “1” on the keyboard. They were informed that some trials (catch trials) did not contain a “jump” and that they were not required to press a button in such a case. This control task consisted of 24 jump trials and eight catch trials.

Questionnaires: Finally, all participants also completed the adult Developmental Coordination Checklist (ADC, Kirby et al., 2010), The Kinesthetic and Visual imagery questionnaire (KVIQ)‐20, using dominant and non‐dominant sides where applicable (Malouin et al., 2007) and the Autism Quotient (Baron‐Cohen et al., 2001; Table 1).

### Analysis

Occlusion paradigm: Responses were removed if reaction times were <150 ms or >3500 ms from the end of the occlusion period as they were unlikely to be meaningful responses. Participants were removed if they showed >33% errors on the control task (1 autistic participant, 3 non‐autistic participants) or >50% misses during the experiment (1 autistic participant). Accuracy of prediction was obtained for each continuation condition by calculating the percentage of correct responses. In order to assess biases in the timing of prediction, the percentage of in time responses was calculated for every continuation condition. As an example, a bias toward slower prediction processes may result in too far behind continuations being incorrectly labeled as in time. Reaction time was not considered a valid measure as participants were not asked to make a speeded response which may have led to more variable reaction times and strategies.

Outlier removal was performed at the group level on the mean responses based on the non‐recursive procedure recommended by Van Selst and Jolicoeur (1994), resulting in the removal of one autistic and one non‐autistic participant for % in time responses. Therefore, there were 19 non‐autistic and 18 autistic participants for accuracy analysis and 18 non‐autistic and 17 autistic for prediction timing biases analysis. Data and analysis scripts for these participants are openly available (Gowen et al., 2022).

Analysis was conducted in Rstudio (Version 1.1.463). As assumptions of normality were violated for accuracy of prediction, a non‐parametric Mann–Whitney test was used to compare between groups and Friedman and Wilcoxon pairwise comparisons used to compare across continuations. Effect sizes were calculated using Mann Whitney and Wilcoxon pairwise testing and Kendall's W for Friedmans tests. In time responses were analyzed using a repeated measures ANOVA with factors of continuation x condition x group and follow up t tests with either generalized eta square (ges) or Cohens d effect sizes provided. Where Maunchlys test of sphericity was violated, Greenhouse Geisser corrections were performed. If data did not show equality of variances (Levenes test <0.05), Welch adjusted t stat was used. We also calculated the Bayes factor using the Bayes Factor package in r (Morey & Rouder, 2018) for in time responses as they were normally distributed. The Bayes factor (BF10) expresses the relative evidence in support of the alternative hypothesis compared to the null hypothesis, given the observed data. BF10 ⩾ 3 suggests increasingly strong evidence in favor of the alternative hypothesis, while BF10 ⩽ 0.33 suggests increasingly strong evidence in favor of the null. When BF10 = 1, the data do not support either hypothesis (Wagenmakers et al., 2018).

For in time responses we performed planned polynomial contrasts to test the trend model (linear, quadratic or cubic) that best described the performance of each group (Brich et al., 2018; Diersch et al., 2012). This was to check whether responses followed the expected inverted u‐shape (quadratic function) with a greater percentage for the in‐time continuation and dropping for the too far ahead/behind conditions. Lack of a significant quadratic function, or the presence of another function would indicate a different pattern of discrimination between in time and other continuations.

Correlations with questionnaires: Depending on distribution, Pearson's or Spearmans correlations were performed between mean prediction accuracy for each participant and the KVIQ, the familiarly scores and the ADC checklist. The mean prediction accuracy across the continuations was chosen to provide an overall measure of accuracy and it did not seem appropriate to choose the accuracy for one particular occlusion continuation.

Visual attention: To provide a general assessment of visual attention to the task, an area of interest including the area of screen in which the video was shown was created using SR Research Data Viewer software and the percentage of time each participant spent in this area calculated. Group percentages were compared using *t* tests or non‐parametric equivalents. Note that we were unable to perform additional eye tracking analysis as the experiment was not constructed to study predictive gaze: Many of the actions in the videos did not involve large spatial movements which would have been necessary to measure predictive eye movements and no synchronization between eye‐tracking and stimulus presentation devices was implemented so we could not overlay eye position over the videos.

Control task: For the control task, the percentage of correct detection of “jump” trials was compared between groups using t tests or non‐parametric equivalents.

## RESULTS

### Prediction accuracy

**FIGURE 2:**
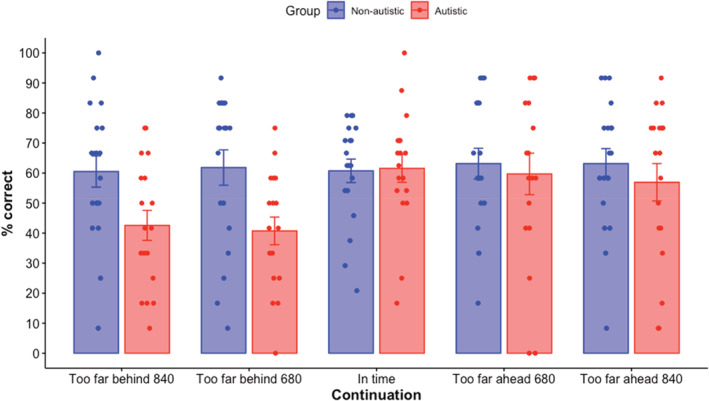
Mean prediction accuracy (% correct) for autistic and non‐autistic groups across the different continuations. Standard error bars are shown. Dots represent individual participants

The data did not appear normally distributed (left skewed) and Shapiro–Wilk testing revealed non‐parametric distributions (*W* = 0.970, *p* < 0.001). Therefore, non‐parametric tests were used—the Mann–Whitney test to assess group differences, followed by Friedman and Wilcoxon tests to assess the effect of continuation in each group separately. Examining interactions was not possible due to the non‐parametric nature of the data. A Mann–Whitney test revealed that the non‐autistic group (mean = 61.9%, std = 21.6) were significantly more accurate than the autistic group (mean = 52.3%, std = 24.7; *U* = 5133.5, *p* = 0.02, *r* = 0.39; Figure 2).

A Friedman test for the autistic group revealed there was a significant effect of continuation (χ2 [4] = 15.7, *p* = 0.003, *W* = 0.22). Wilcoxon pairwise comparisons showed that compared to in time (mean = 62%, std = 20), the proportion of correct responses was lower for continuations that were too far behind by 680 ms (mean = 41%, std = 20; *p* = 0.003, *r* = 0.70) and 840 ms (mean = 43%, std = 21; *p* = 0.002, *r* = 0.73; Figure 2). There were no other significant differences between the continuations (*p* ≥0.03, Bonferroni significance level <0.005). A Friedman test for the non‐autistic group indicated that there were no significant effects of continuation (χ2 [4] = 1.67, *p* = 0.80, *W* = 0.02).

### Prediction timing biases

In order to assess timing biases, the percentage of in time responses was compared between the continuation conditions. In the absence of any timing biases, the % of “in time” responses should be maximal for the in time continuation with few in time responses given for the other continuations. Biases would appear when in time continuations are incorrectly labeled as too far ahead/behind due to participants confusing the too far behind or ahead occlusion with in time.

**FIGURE 3:**
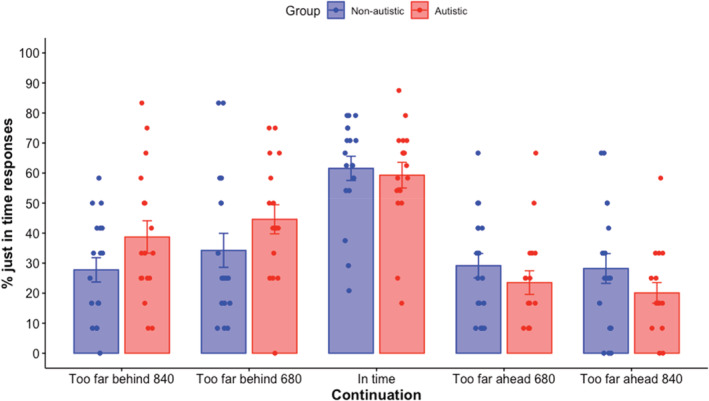
Prediction timing biases (% in time responses) for autistic and non‐autistic groups across the different continuations. Standard error bars are shown. Dots are individual participants

The data appeared normally distributed, confirmed with Shapiro Wilkes testing (*W* = 0.99; *p* = 0.24). A mixed ANOVA (group × continuation) showed that there was a main effect of continuation [*F*(2.72) = 23.26; *p* < 0.001; ges = 0.33; BFinc = 4.889e + 10], no main effect of group [*F*(1.33) = 0.09; *p* = 0.76; ges = 0.0008; BFinc = 0.18] and no interaction ([*F*(2.72) = 2.23; *p* = 0.11; ges = 0.05; BFinc = 0.34] (Figure 3). Post hoc paired *t* tests indicated that when corrected for multiple comparisons (Tukey method) there were significantly more “in time” responses for the in time continuation (60.4%) compared to all other continuations (too far behind by 840 ms (33.2%; *t* = −5.67, *p* < 0.001, *d* = −0.50); too far behind by 680 ms (39.4%) (*t* = −4.47, *p* = 0.001, *d* = 0.40); too far ahead by 840 ms (24.2%; *t* = 10.4, *p* < 0.001, *d* = 0.92); too far ahead by 680 ms (26.3%; *t* = 9.57, *p* < 0.001, *d* = 0.85). In addition, there were significantly more “in time” responses for too far behind 680 ms compared to too far ahead 840 ms (*t* = 3.19, *p* = 0.03, *d* = 0.28). All other comparisons were not significant (*t* < 2.6; *p* > 0.09).

The trend analysis revealed a significant quadratic trend for both autistic (*F*[1.80] = 09.65, *p* = 0.003) and non‐autistic (*F*[1.84] = 10.24, *p* = 0.002) participants. This indicated that in time responses were correctly more frequent for the in‐ time continuation and leveled out at both too far behind and too far ahead continuations. However, only the autistic group showed a significant linear trend (autistic: *F*(1.80) = 21.02, *p* < 0.001; non‐autistic: *F*(1.80) = 0.54, *p* = 0.47), indicating that in time responses linearly increased from too far behind to in time continuations (Figure 3).

### Questionnaire data

Data for familiarity and KVIQ‐V were non‐normally distributed (Shapiro–Wilk test, *p* = 0.003 and 0.02, respectively), while data were normally distributed for KVIQ‐K and the DCD checklist, therefore Mann–Whitney tests were used for the former two. The autistic group reported being significantly less familiar with the actions and had significantly more coordination difficulties on the DCD checklist (Table 2). The groups showed equivalent results on the KVIQ‐V and KVIQ‐K (Table 2).

**TABLE 2:** Mean and median scores for the different questionnaires in the two participant groups, along with results of the statistical comparison

	Autistic group	Non‐autistic group	Comparison
Familiarity	Mean = 7.14 ± 1.97 Median = 7.23	Mean = 8.64 ± 1.18 Median = 8.92	*U* = 256.5, *p* = 0.009, *r* = −0.43
KVIQ‐V	Mean = 56.4 ± 23.0 Median = 57	Mean = 68.2 ± 12.3 Median = 71	*U* = 219, *p* = 0.14, *r* = −0.24
KVIQ‐K	Mean = 47.2 ± 24.0 Median = 46	Mean = 47.2 ± 14.5 Median = 48	*t* = −0.001, *p* = 1.0, *d* < 0.001
DCD checklist	Mean = 64.2 ± 10.7 Median = 65	Mean = 27.6 ± 12.2 Median = 26	*t* = −9.68, *p* < 0.001, *d* = 3.18

Spearmans correlation revealed no significant correlations between mean prediction accuracy and familiarity (autistic: *r* = 0.11, *p* = 0.67; non‐autistic: *r* = −0.24, *p* = 0.33) and mean prediction accuracy and KVIQ_V (autistic: *r* = −0.17, *p* = 0.5; non‐autistic: *r* = −0.40, *p* = 0.09). Pearsons correlations revealed no significant correlations between mean prediction accuracy and KVIQ_K (autistic: *r =* −0.09, *p* = 0.73; non‐autistic: *r* = −0.47, *p* = 0.04) and mean prediction accuracy and the ADC checklist (autistic: *r* = −0.04, *p* = 0.88; non‐autistic: *r* = −0.07, *p* = 0.76; Bonferroni significance level <0.01).

### Visual attention and control task

The Shapiro–Wilk test indicated the % dwell time data were not normally distributed (*p* < 0001) so a Mann–Whitney test was used to compare the % of time that autistic compared to non‐autistic participants viewed the videos. There were no significant group differences (autistic: mean = 96%, std = 6%; non‐autistic: mean = 99%, std = 3% *U* = 166, *p* = 0.66, *r* = 0.07).

For the control task, the data were non‐normally distributed (Shapiro–Wilk *p* < 0.001), so Mann Whitney testing was used for group comparisons. Autistic (mean = 95% correct) and non‐autistic (mean = 91% correct) were equally able to detect the jumpy trials (*U* = 146, *p* = 0.43, *r* = 0.13) and both groups correctly withheld responses to all no jump trials.

## DISCUSSION

This study used a well‐established occlusion paradigm to test whether autistic adults are able to predict the pace of unfolding actions to a similar degree as non‐autistic participants. The autistic participants were less accurate than the non‐autistic participants, particularly when the action continued too far behind. This is consistent with the trend analysis where only the autistic group showed a linear trend, reflecting the gradual increase of in time responses from the too far behind to in time continuations. Thus, the autistic group showed less ability to discriminate between the intime continuation and the too far behind 680 and 840 conditions, although it should be noted that the interaction between group and condition was not significant. Previous work examining action prediction in autistic individuals has focused on the integrity of contextual cues, such as changes to the surroundings or social cues and shown that they make less use of this prior contextual knowledge (Amoruso et al., 2019; Chambon et al., 2017). Our findings highlight that predicting actions using kinematic information may also be difficult for autistic individuals, potentially contributing to challenges experienced across a range of social settings where prediction based on kinematics is important (Ansuini et al., 2015; Becchio et al., 2012). However, findings do warrant future replication in view of the non‐significant group x continuation interaction for % in time responses.

A number of lines of evidence suggest that action prediction ability during occlusion paradigms relies on motor simulation processes. For example, action prediction ability improves with increasing motor experience of the observed action (Stapel et al., 2016) and is negatively impacted by concurrent motor execution (Springer et al., 2011), disruption to premotor areas using Transcranial Magnetics Stimulation (Stadler, Ott, et al., 2012) and the presence of neurological motor impairments (de Wit & Buxbaum, 2017). Previous studies in non‐autistic participants using similar occlusion tasks have observed a delay in simulation so that actions that have been moved back in time, appearing too far behind are mistaken as being in time (Brich et al., 2018; Prinz & Rapinett, 2008; Sparenberg et al., 2012). This is thought to be due to a switch cost between online action perception processes and offline action simulation processes during the occluded period (Prinz & Rapinett, 2008; Sparenberg et al., 2012). A similar pattern of confusing too far behind responses with in time responses was observed in our autistic group suggesting that they are slower to start actively simulating during the occlusion period. In contrast, the non‐autistic group in our study were consistently accurate across the continuations and only showed a quadratic function, indicating that in time continuations were correctly identified and less confused with too far ahead/behind continuations.

The lack of evidence for a delay in simulation for our non‐autistic participants is likely to be due to the larger window over which the continuations were placed in our experiment. Previous studies tested continuations within a window of <±500 ms, whereas our study tested a window of ±840 ms, with the smallest continuation (680 ms) falling outside 500 ms. Similarly, to Diersch et al. (2012), who compared an older to a younger group, we chose a larger window to ensure the task would not be too difficult for the participants. Therefore, it is highly likely that any delay in simulation for the non‐autistic participants is smaller than the 680 ms continuation and participants are able to differentiate the too far behind continuation correctly. Importantly, the finding that the autistic group still show less accurate responses for the too far back conditions despite the larger continuation window highlights a significant delay in simulation processing. Difficulties with switching from online action perception to covert stimulation links with cognitive flexibility anomalies in autism such as task switching and set shifting (Demetriou et al., 2018; Uddin, 2021; Watanabe et al., 2019). It is not possible to distinguish whether simulation is delayed due to switching between online action perception and simulation or generally slower in the autistic group and future work should aim to test this as in previous non‐autistic studies (Prinz & Rapinett, 2008; Sparenberg et al., 2012). Eye tracking may also add valuable knowledge as to whether participants were using a predictive strategy when watching the videos or during the occlusion period (Bache et al., 2017) and its absence is a limitation of the current study. This could be explored using a more limited set of spatially extended actions. Such lines of enquiry will be important for understanding whether action prediction difficulties may be explained by predictive coding accounts of action perception, a topical theme in autism research (Parr et al., 2018; Pellicano & Burr, 2012; Van de Cruys et al., 2014).

Delayed or slower simulation during this prediction task fits with previous evidence of altered simulation in autistic groups suggested by findings from hand or body rotation tasks (Chen et al., 2018; Conson et al., 2013, 2015, 2016). While some findings suggest typical use of motor imagery in autistic groups since they are faster to judge comfortable versus physically awkward hand postures (Chen et al., 2018; Conson et al., 2016), this biomechanical effect has not always been observed in the autistic group (Conson et al., 2013), Moreover, one study found the autistic group were more affected by the posture of their own arm, suggesting difficulty in separating covert motor simulation from overt motor activity. In addition, Chen et al. (2018) reported longer reaction times for judging hand stimuli in the autistic group, consistent with a slower onset of simulation. Nevertheless, our results on the KVI‐Q indicate that our participants rated the vividness of their motor imagery at a similar level as the non‐autistic group. Overall, these previous findings, together with our results on the action prediction task, suggests that autistic individuals may use motor simulation, but that this is less efficient. Interestingly, there were also no significant correlations in either group between the KVIQ and prediction accuracy, suggesting that the tasks tap into different aspects of simulation, with the KVIQ involving generation of a motor image, while action prediction involving maintenance or manipulation of the simulated image (Kraeutnera et al., 2020). However, it should be noted that this secondary exploratory analysis was only powered to detect large effect sizes, so it is possible that small‐medium effect sizes might not be detected. Further research is required to understand what components of action prediction and motor imagery ability are linked in both autistic and non‐autistic individuals.

However, it should also be considered whether the autistic group were using simulation to perform the prediction task. An additional mechanism that may contribute to action prediction is visual extrapolation of the movement through local motion and velocity cues, involving visual rather than motor mechanisms (Stadler, Springer, et al., 2012; Vannuscorps & Caramazza, 2016; Zhu & Bingham, 2014). Indeed, previous research indicates intact visual extrapolation in autistic children using an occlusion paradigm where participants are required to indicate the terminal position of an occluded moving non‐human target such as a car (Tewolde et al., 2018). Furthermore, basic motion processing for non‐human stimuli are frequently reported as similar or enhanced in autistic and non‐autistic groups (Foss‐Feig et al., 2013; Manning et al., 2015). Therefore, it is possible that autistic individuals used visual extrapolation in our prediction task which could be tested in future work using action‐perception interference with concurrent motor execution paradigms (Springer et al., 2011). A further consideration is whether some participants used memory processes to perform the task, as they saw the videos once before the experiment phase and then the videos were repeated twice during the experiment phase. Consequently, some participants may have remembered the speed of a particular action in the video or the last frame before occlusion and compared that following the occlusion period. However, this would be a difficult strategy as the videos are quite long (1 min) and complex and participants would not know which parts were going to be occluded until the experiment part. We also asked participants whether they used a particular strategy and no participants mentioned the use of memory.

Consistent with evidence of motor coordination difficulties, the autistic group had significantly higher scores on the ADC and were also less familiar with the actions. As mentioned earlier, action prediction ability has been shown to be negatively impacted by motor execution and motor impairments but also to improve with motor experience so one might expect that poorer motor coordination and less familiarity with the actions might be related to action prediction ability in our task. In addition, less familiarity with the actions could lead to both lower motor and visual experience of the actions (Calvo‐Merino et al., 2006). However, no significant correlations were found, although note the earlier point about correlations being powered for large effect sizes only. Previous work has found correlations between autistic motor ability and performance on action discrimination or identification tasks (Lindor et al., 2019; Price et al., 2012), but these studies used motor tasks rather than a questionnaire. It is possible that autistic participants were more conservative when rating familiarity and that objective motor tasks that are more similar to the actions depicted in the videos may be more sensitive. Combining motion tracking with motor tasks would also provide information on the kinematics of the executed movements, such as jerkiness. As autistic individuals tend to produce more jerky movements (Cook, 2016), it would be valuable to test whether levels of jerkiness correlate with action prediction ability. This would indicate whether action prediction errors directly relate to expectations built up from the participants own movement patterns. Finally, a narrower window of continuations may also bring out larger individual differences that may relate more strongly to motor ability or familiarity.

## CONCLUSION

In summary, autistic individuals were less accurate than non‐autistic individuals at correctly predicting the pace of an unfolding action using an occlusion paradigm. This pattern appeared to be due to the autistic group confusing actions that had been moved too far behind with in time actions, suggesting delayed or slower simulation processes. Future work should aim to replicate this finding, differentiate delayed from slowed simulation ability and investigate the influence of motor ability and familiarity individuals have with the actions.

## ETHICS STATEMENT

The study was approved by The University of Manchester Research Ethics Committee.

## Data Availability

The data and analysis scripts that support the findings of this study are openly available in Open Science Framework at https://osf.io/mwgyq/ [DOI 10.17605/OSF.IO/MWGYQ] (Gowen et al., 2022).
